# Machine learning reveals that structural features distinguishing promiscuous and non-promiscuous compounds depend on target combinations

**DOI:** 10.1038/s41598-021-87042-z

**Published:** 2021-04-12

**Authors:** Christian Feldmann, Jürgen Bajorath

**Affiliations:** Department of Life Science Informatics and Data Science, B-IT, LIMES Program Unit Chemical Biology and Medicinal Chemistry, Rheinische Friedrich-Wilhelms-Universität, Friedrich-Hirzebruch-Allee 6, 53115 Bonn, Germany

**Keywords:** Drug discovery, Chemistry, Mathematics and computing

## Abstract

Compounds with defined multi-target activity (promiscuity) play an increasingly important role in drug discovery. However, the molecular basis of multi-target activity is currently only little understood. In particular, it remains unclear whether structural features exist that generally characterize promiscuous compounds and set them apart from compounds with single-target activity. We have devised a test system using machine learning to systematically examine structural features that might characterize compounds with multi-target activity. Using this system, more than 860,000 diagnostic predictions were carried out. The analysis provided compelling evidence for the presence of structural characteristics of promiscuous compounds that were dependent on given target combinations, but not generalizable. Feature weighting and mapping identified characteristic substructures in test compounds. Taken together, these findings are relevant for the design of compounds with desired multi-target activity.

## Introduction

Polypharmacology results from the in *vivo* modulation of multiple targets^1–3^, which is often required for effective therapeutic intervention of multi-factorial pathologies such as cancer or neurodegenerative diseases^3–5^. While polypharmacology can principally be provoked through drug combination therapy, the administration of multi-target (promiscuous) drugs is generally preferred^4,5^. As a prerequisite of polypharmacology, multi-target activity of small molecules is based upon the ability to form “pseudo-specific” interactions with different targets. At first sight, such “selectively nonselective” interactions^6^ are paradoxical and currently only little understood at the atomic level of detail. Yet, rationalizing such interactions and their molecular determinants will be critical for learning how to design compounds with desired multi-target activity, which is a highly topical issue in medicinal chemistry^7–9^.

Systematic analysis of X-ray structures of complexes formed by proteins from different families with promiscuous compounds has revealed that about half of these ligands, regardless of whether they were rigid or flexible, bound with similar conformations to multiple targets, but formed different target-dependent interaction patterns in their binding sites^10^. On the other hand, promiscuous compounds interacting with functionally distinct targets often displayed different binding modes^11^. Furthermore, a given multi-target ligand might adopt a similar binding mode interacting with some of its targets and very different ones with others^11^. Hence, binding characteristics of promiscuous compounds varied greatly and were not generalizable.

It is currently also unknown whether multi-target compounds share particular structural features that are responsible for their ability to interact with different targets. While structure–activity relationship (SAR) analyses have thus far not identified common structural signatures of multi-target compounds, indirect evidence for the existence of such features has been provided through machine learning (ML). Different ML models were trained to systematically distinguish between promiscuous and non-promiscuous compounds from medicinal chemistry with activity against related or unrelated targets on the basis of chemical structure. These models reached more than 70% accuracy in predicting compounds with multi-target activity^12,13^. Equivalent results were obtained when distinguishing between multi- and single-target compounds from biological screens that were extensively tested in comparably large numbers of assays^14^. For these screening compounds, negative assay results were available such that groups of multi- and corresponding single-target compounds could be assembled, thus ensuring data completeness for promiscuity predictions^14^. In all cases, the accuracy of the predictions strongly depended on (similarity-based) nearest neighbor (NN) relationships between multi- or single-target compounds^12–14^. When compounds forming NN relationships were removed from training sets, prediction accuracy was significantly reduced, but not abolished. Many single- and multi-target compounds were found to form separate analog series and only few series were identified that combined single- and multi-target compounds^14^. Hence, many promiscuous compounds were more similar to each other than to non-promiscuous compounds and vice versa.

Taken together, these findings raised a key question for rationalizing the basis of compound promiscuity. Do structural features exist that generally characterize promiscuous compounds, regardless of the targets they are active against, or is the molecular basis of promiscuity determined “locally”, i.e., through structural characteristics that depend on individual targets? This question is comprehensively investigated in the following.

## Results

### Study concept

**Figure 1:**
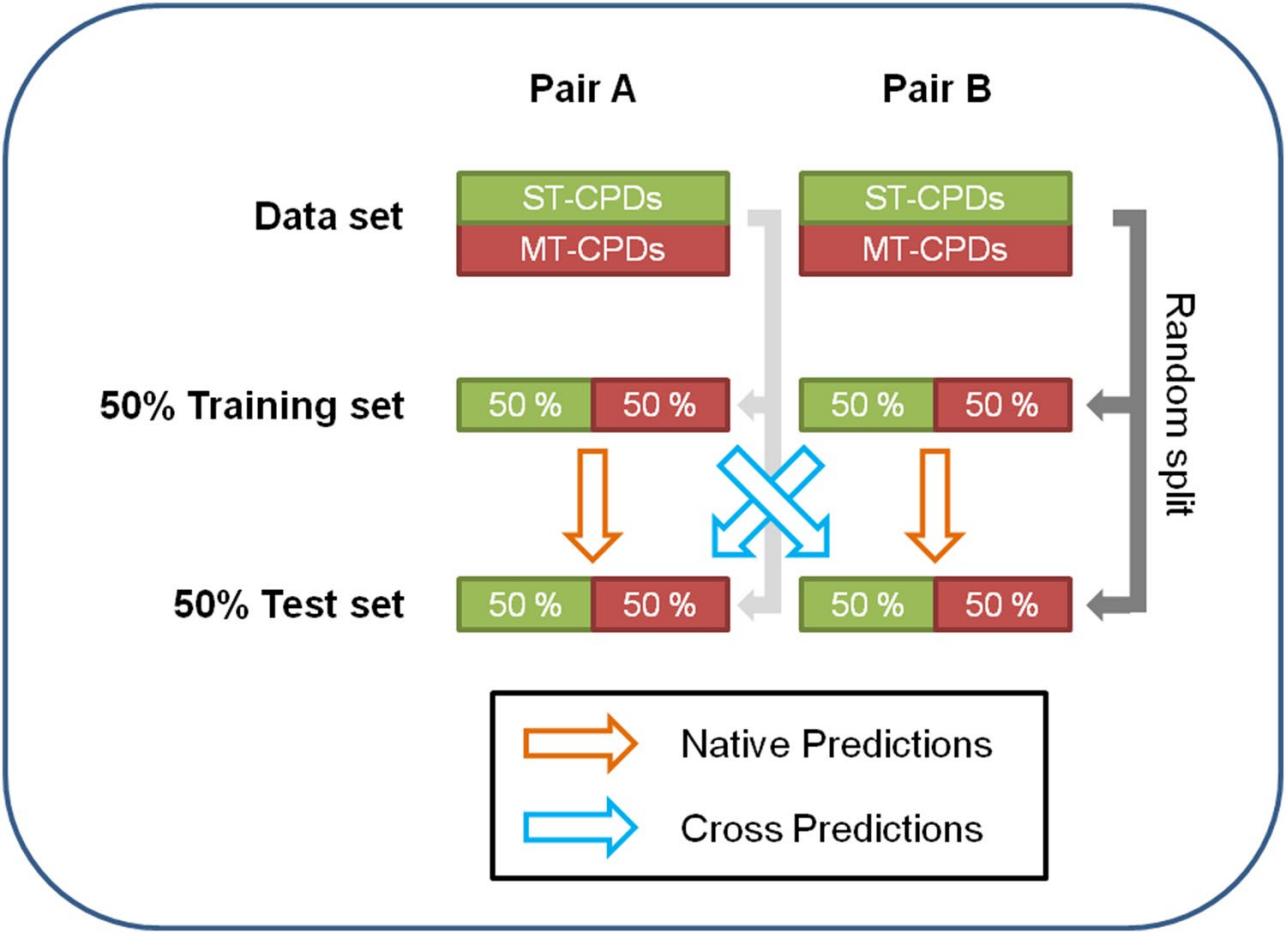
Native and cross predictions. The schematic illustrates our ML approach. For each target pair, MT- and ST-CPDs were evenly divided into training and test sets. Different ML models were derived and applied to predict test sets of the same target pair (native prediction) and different target pairs for which the model was not derived (cross predictions). In each case, this procedure was repeated 10 times with test and training sets of different composition.

To address the key question whether or not characteristic features might generally be shared by promiscuous compounds, we have devised a unique test system for diagnostic machine learning. From compounds with known activity against current pharmaceutical target proteins (in the following, the term target exclusively refers to proteins), data sets were systematically assembled that consisted of at least 50 compounds with activity against target A (single-target compounds; ST-CPDs), 50 compounds active against target B (ST-CPDs), and 100 compounds active against A + B (dual-target compounds, DT-CPDs). Accordingly, each data set represented a unique target combination and DT-CPDs represented prototypic data set-specific promiscuous compounds. For each data set, different ML models were generated on the basis of chemical structure to distinguish between DT- and corresponding ST-CPDs (native predictions). Then, each target pair-specific classification model was used to systematically predict the test sets of all other target pairs (cross-pair predictions). Figure 1 schematically illustrates the approach.

The underlying rationale was the following: If structural features characteristic of promiscuous compounds exist, native ML predictions should be successful. Moreover, if characteristic features were “global” in nature, i.e., common to many promiscuous compounds, cross-pair predictions should succeed, in principle. By contrast, if features characteristic of promiscuous compounds were “local” in nature, i.e. confined to individual target combinations, cross-pair predictions could not be generally successful but should mostly fail. Hence, using this test system and evaluation strategy, it was possible to rigorously examine the key question concerning characteristic features of promiscuous compounds. In this context, ML served as a diagnostic approach, which means that positive and negative prediction outcomes were considered as an indicator for the presence or absence of structure-promiscuity relationships.

### Data sets and model building

We identified a total of 170 unique target pair-based compound data sets comprising at least 100 MT- and 100 (50 + 50) corresponding ST-CPDs. These data sets covered a total of 137 distinct targets. Most data sets (157) involved targets from the same protein family, while 13 sets involved targets from different families (Supplementary Table S1). Data sets were balanced in size relative to the limiting number of ST- or DT-CPDs. For example, if 150 ST-CPDs and 200 ST-CPDs were available for target A and B, respectively, and 110 DT-CPDs, the final size of the data set for this target combination was 220 compounds (55 + 55 ST- and 110 DT-CPDs).

For each data set, random forest (RF), support vector machine (SVM), and k-NN classification models were generated on the basis of randomly selected 50% of the compounds and tested on the remaining 50%. Models were built using different structural fingerprint representations of compounds and subjected to nested cross-validation for hyperparameter optimization (see Supplementary Methods). Predictive performance of each model was assessed as a mean over 10 independent trials using different performance measures including balanced accuracy (BA), the F1 score, Matthews correlation coefficient (MCC), recall, and precision (see the Methods section).

### Native and cross-pair predictions

**Figure 2:**
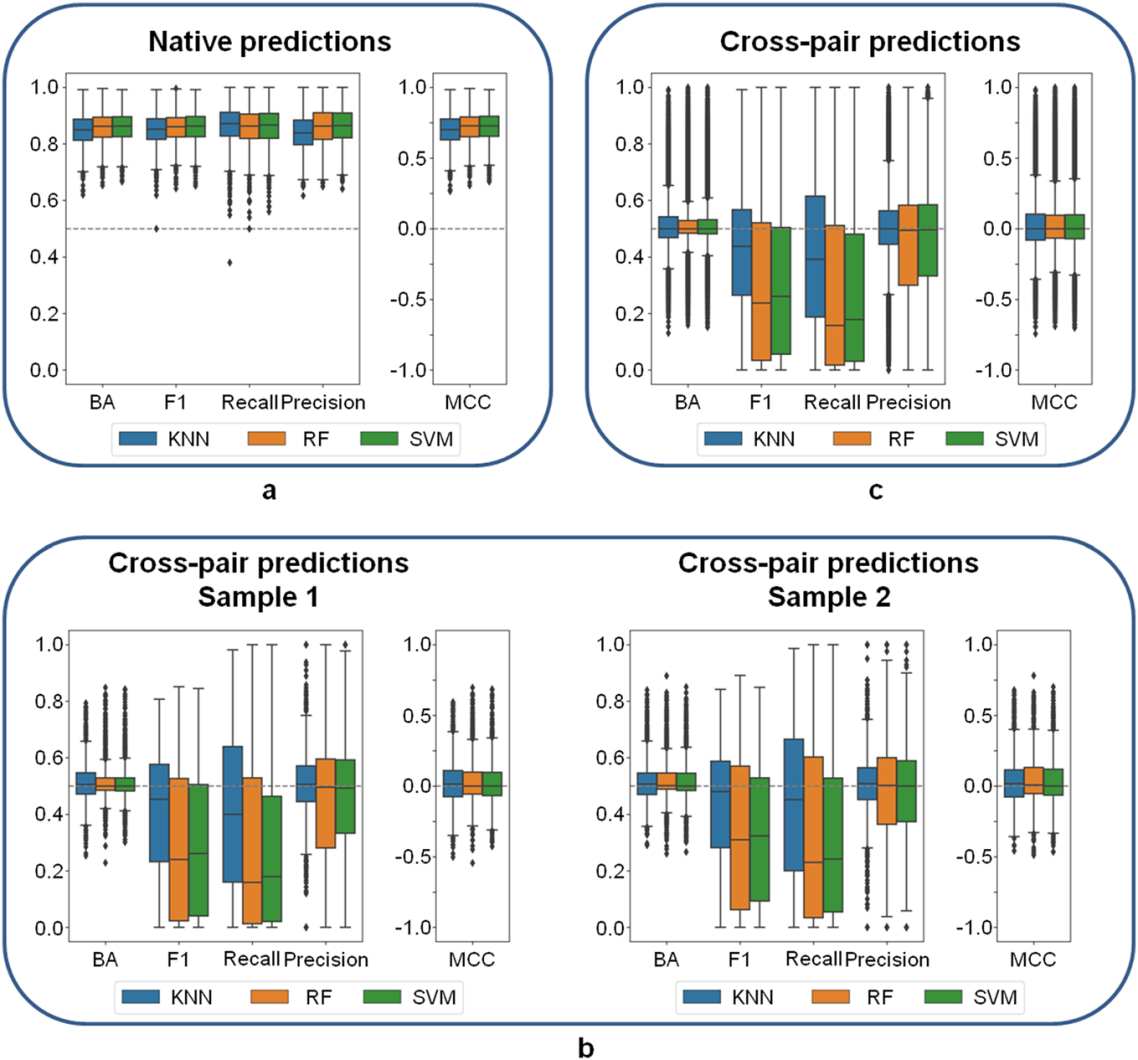
Model performance. Boxplots report distributions of ML model performances on the basis of different measures (KNN: blue, RF: orange, SVM: green). In a box plot, a distribution is represented by its maximum (upper whisker), upper quartile (upper boundary of the box), median (horizontal line in box), lower quartile (lower boundary of the box) and its minimum (lower whisker). Individual values classified as statistical outliers are shown as diamonds. (**a**) Models were evaluated on test sets for the same target pairs they were trained on (native model predictions). (**b**) Shown are two representative examples of models that were evaluated on test sets from different target pairs (cross-pair predictions). (**c**) Model performance is reported over all 28,730 cross-pair predictions.

Figure 2 summarizes the results of systematic native and cross-pair predictions. Figure 2a shows that ML models generally distinguished DT- and ST-CPDs with high accuracy greater than 80% on the basis of different performance measures and median MCC values ~ 0.75, with the exception of limited numbers of statistical outliers.

Hence, native predictions using models derived for each pair were overall accurate, confirming the presence of distinguishing structural features. Notably, k-NN classifiers approached or met the performance of RF and SVM models, highlighting the relevance of NN relationships for target pair-based predictions.

We next compared native and cross-pair predictions. To enable direct comparison, we initially drew 10 random samples of 170 cross-pair predictions from their large pool (the complete matrix of cross-pair predictions contained 28,730 unique entries). Figure 2b shows the results for two representative examples (results for the remaining eight samples are shown in Supplementary Fig. S1). The results obtained for all samples were very similar. With the exception of some statistical outliers, prediction accuracy was consistently random (or worse than random on the basis of the F1 and recall measures). The same result was obtained for the complete matrix of cross-pair predictions, as shown in Fig. 2c. On the basis of all performance measures, median prediction accuracy corresponded to random (or worse than random) predictions. As an additional control, we extracted cross-pair subsets from the matrix where targets from both pairs belonged to the same family (5030 cross-pairs) or each pair to a different family (19,462 cross-pairs) and separately analyzed predictions for these subsets. For cross-pairs from different families, prediction accuracy was random and for cross-pairs from the same family—where one might anticipate potential target correlation effects—prediction accuracy was marginally better than random (Supplementary Fig. S2). Hence, even for related target pairs, most models were not transferable. Finally, for the 13 pairs of targets from different families (reported in Supplementary Table S1), native predictions were highly accurate, whereas cross-pair predictions also yielded random (or in part much worse than random) accuracy in most cases (Supplementary Fig. S3).

Taken together, the results clearly demonstrated that in the vast majority of cross-pair evaluations, target pair-based ML models were not predictive, thus ruling out the presence of structural features that generally distinguished between DT- and ST-CPDs.

### Feature weighting and mapping

**Figure 3:**
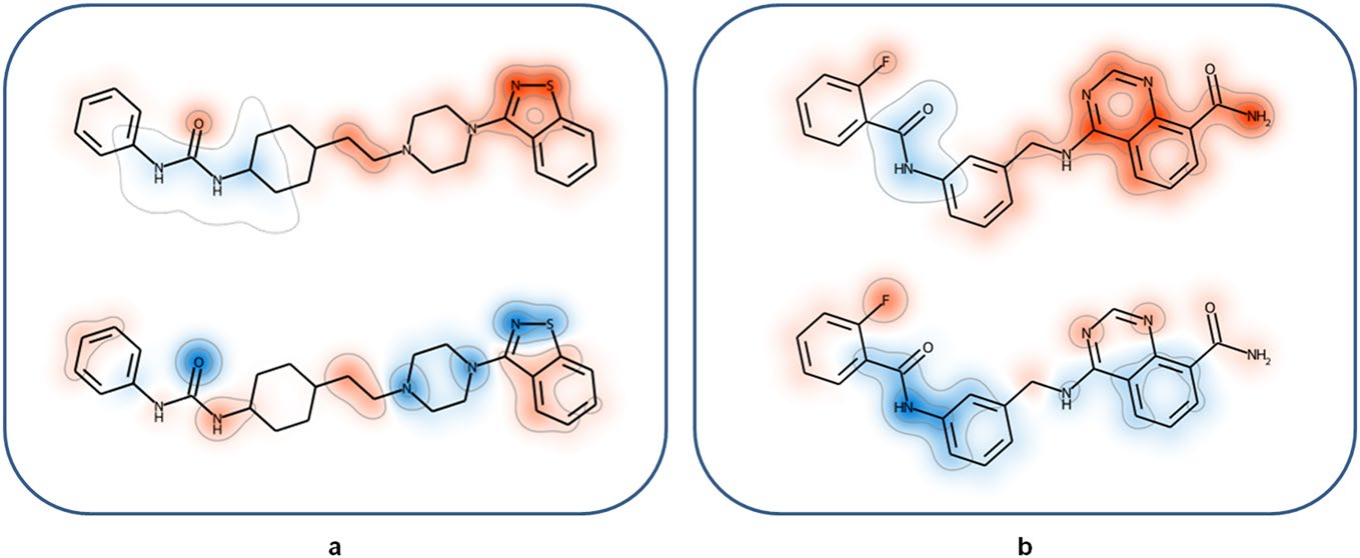
Feature weight mapping. Shown are SVM weights of individual features mapped to corresponding atoms. A Gaussian function proportional to the atom weight was placed on each atom position. The resulting height profile was visualized using a red color gradient for positive values, i.e., weights associated with DT activity (responsible for correct predictions), and a blue color gradient for negative values (weights associated with ST activity; incorrect predictions). (**a**) Depicted is a DT-CPD active against the 5-hydroxytryptamine receptor 1A and dopamine D2 receptor. At the top and bottom, feature contributions from the native classifier and a model derived for another target pair (glucocorticoid receptor, progesterone receptor) are depicted, respectively. (**b**) A DT-CPD with activity against Aurora kinase A and B is shown. At the top and bottom, feature contributions from the native classifier and a model derived for a different target pair (ocxytocin receptor, vasopressin V1a receptor) are shown.

For SVM models, structural features determining the predictions can be directly identified through support vector weighting and mapping of atoms forming highly weighted features (see the Methods section). Positively and negatively weighted features contribute to the prediction of DT- and ST-CPDs, respectively. Figure 3 shows exemplary results. In Fig. 3a, a DT-CPD with activity against the 5-hydroxytryptamine receptor 1A and dopamine D2 receptor is shown. At the top, feature contributions from the native classifier are highlighted that correctly predicted this test compound. Mapped positive feature weights (red) delineated a substructure comprising the benzisothiazole and piperazine rings that determined the correct prediction. This substructure was shared by other DT-CPDs for this target pair. Only very small negative feature weight contributions were detected in the vicinity of the urea moiety on the other side of the compound. At the bottom, highly weighted features from an incorrect cross-pair prediction of this DT-CPD by an SVM model derived for a different receptor pair are shown. Here, feature weights were of lesser magnitude than observed for the correct predictions and the distribution of the associated features was distinct, not recognizing the substructure responsible for the correct prediction, but assigning negative feature weights to this molecular region. Cross-pair predictions displayed a general tendency to lack highly weighted features delineating coherent substructures. Instead, positive and negative weights of limited magnitude were often scattered across test compounds, thus indicating that the model did not recognize signature features it learned during native training. Figure 3b depicts another exemplary DT-CPD with activity against the closely related Aurora kinases A and B. The corresponding SVM model accurately distinguished between DT- and ST-CPDs available for this target pair. At the top, highly weighted positive features from the native model clearly identified the substituted quinazoline substructure to be critically important for the correct prediction. By contrast, as shown at the bottom, a model derived for a pair of G protein coupled receptors assigned non-decisive low positive and negative weights to the quinazoline substructure, but higher negative weights to the anilide substructure, leading to an incorrect prediction. The examples illustrate that feature weight mapping can identify substructures that determine correct predictions of DT-CPDs. Such substructures can be further considered and explored as characteristic promiscuity signatures.

## Conclusion

Rationalizing origins of multi-target activity of small molecules is of prime relevance for drug discovery. However, our current understanding of molecular features that enable compounds to “pseudo-specifically” interact with multiple targets is rather limited. In particular, it is currently unclear whether molecular signatures exist that generally characterize promiscuous compounds. Such features have been elusive so far, if they exist at all. Our current investigation was conceptualized to conclusively answer this question. It was catalyzed by our previous observation that many multi-target compounds were more similar to each other than to corresponding single-targets compounds and vice versa. The test system we devised enabled the use of ML on the basis of chemical structure to distinguish between DT- and corresponding ST-CPDs over 170 qualifying target combinations with available high-confidence activity data (and at least 100 DT-CPD to enable statistically meaningful assessment). While the majority of qualifying target pairs originated from the same protein families, most cross-pair predictions involved target pairs from different families. Hence, the calculations tested for the presence of “local” or “global” structural features characteristic of promiscuous compounds. The results we obtained were exceptionally clear. Native predictions consistently distinguished between DT- and corresponding ST-CPDs with high accuracy. By contrast, systematic cross-pair predictions essentially failed (with few exceptions due to target correlation, as expected). Thus, taken together, these findings provided compelling evidence that characteristic features of DT-CPDs that set them apart from ST-CPDs existed and depended on the target combinations they were active against (local features). By contrast, there were no detectable (global) features that generally characterized compounds with multi-target activity. Weighting and mapping of features from target pair-dependent SVM classifiers highlighted exemplary substructures in DT-CPDs that determined correct predictions. Such substructures can be further considered as potential signatures in multi-target ligand design. In practice, medicinal chemistry efforts towards polypharmacology predominantly focus on the generation of compounds with desired dual-target activity. To these ends, the presented strategy can be adapted. More target pairs from different families can be generated by lowering the number of required DT-CPDs. As long as a meaningful diagnostic ML model can be generated for a target combination of interest, features characterizing DT-CPDs can likely be identified and further explored.

## Methods

A methods summary is presented herein. Methodological details, data descriptions, programs, and calculation parameters are provided as Supporting Information.

Bioactive compounds with available high-confidence activity data (see Supplementary Methods) were extracted from ChEMBL (version 26)^15^. Target protein families were defined according to the UniProt classification^16^.

Compound classification models were trained using the RF^17^ and SVM^18^ algorithm. In addition, k-NN classifiers were built. The models were derived using different structural fingerprint representations and 10 cross validation trials (see Supplementary Methods). Models discussed herein were generated on the basis of standard atom environment fingerprint representations^19^.

To evaluate the predictions, the following performance measures were applied including balanced accuracy (BA)^20^, Matthew’s correlation coefficient (MCC)^21^, F1 score^22^, precision, and recall.

$$\begin{aligned} {\text{BA}} & = \frac{1}{2}\left( {{\text{TPR}} + {\text{TNR}}} \right) \\ {\text{MCC}} & = \frac{{{\text{TP}} \times {\text{TN}} - {\text{FP}} \times {\text{FN}}}}{{\sqrt {\left( {{\text{TP}} + {\text{FP}}} \right)\left( {{\text{TP}} + {\text{FN}}} \right)\left( {{\text{TN}} + {\text{FP}}} \right)\left( {{\text{TN}} + {\text{FN}}} \right)} }} \\ F1 & = 2 \times \frac{{{\text{TP}}}}{{2 {\text{TP}} + {\text{FP}} + {\text{FN}}}} \\ \end{aligned}$$

TP, TN, FP, and FN stand for true positives, true negatives, false positives, and false negatives, respectively.

Precision reports the proportion of TP among all positive predictions, while recall reports the proportion of recovered TP relative to all positive instances in the data set:

$$\begin{aligned} {\text{Precision}} & = \frac{{{\text{TP}}}}{{{\text{TP}} + {\text{FP}}}} \\ {\text{Recall}} & = { }\frac{{{\text{TP}}}}{{{\text{TP}} + {\text{FN}}}} \\ \end{aligned}$$

For SVM models, a feature weighting method can be applied to identify unique fingerprint features determining positive or negative predictions^23^. After training a SVM-model, its support vectors ***x***^*(i)*^, corresponding Lagrangian multipliers *λ*^*(i)*^, and class labels *y*^*(i)*^ are determined. The weight of feature *d* in the bit vector ***x*** of given compound is then calculated as follows^23^:

$${\text{fc}}\left( {{\mathbf{x}},{\text{d}}} \right) = { }\mathop \sum \limits_{{{\text{support}}\;{\text{vectors}}}} \frac{{{\text{y}}^{{\left( {\text{i}} \right)}} {\uplambda }^{{\left( {\text{i}} \right)}} {\text{x}}_{{\text{d}}}^{{\left( {\text{i}} \right)}} {\text{x}}_{{\text{d}}} }}{{\left\langle {{\mathbf{x}}^{{\left( {\text{i}} \right)}} ,{ }{\mathbf{x}}^{{\left( {\text{i}} \right)}} } \right\rangle + { }\left\langle {{\mathbf{x}},{\mathbf{x}}} \right\rangle - \left\langle {{\mathbf{x}}^{{\left( {\text{i}} \right)}} ,{\mathbf{x}}} \right\rangle }}$$

For feature mapping, feature weights (*fw*) of atoms (*a*) are determined by dividing the weight of each feature by the number of associated atoms (*n*_*Atoms*_), scaled by the number of feature occurrences *n*_*occ*_:

$${\text{fw}}\left( {\text{a}} \right) = { }\mathop \sum \limits_{{{\text{features}}}} \frac{{{\text{fc}}}}{{{\text{n}}_{{{\text{Atoms}}}} {\text{n}}_{{{\text{occ}}}} }}$$

Atom-based feature weight maps were visualized using functions from RDKit^24^.

## Supplementary Information

Supplementary Information.

## Data Availability

All calculations were carried out with public domain data and programs specified in the Supplementary Methods.
