# Supplementary material for: Machine learning reveals that structural features distinguishing promiscuous and non-promiscuous compounds depend on target combinations
Source: Sci Rep. 2021 Apr 12;11:7863. doi: 10.1038/s41598-021-87042-z (PMC8042106; doi:10.1038/s41598-021-87042-z)
Supplement: Supplementary file 1 — Supplementary Information. [file 41598_2021_87042_MOESM1_ESM.pdf]

Supplementary Information

Diagnostic Machine Learning Reveals that Structural Features Distinguishing Promiscuous and Non-Promiscuous Compounds Depend on Target Combinations

Christian Feldmann and Jürgen Bajorath*

Department of Life Science Informatics, B-IT, LIMES Program Unit Chemical Biology and Medicinal Chemistry, Rheinische Friedrich-Wilhelms-Universität, Endenicher Allee 19c, D-53115 Bonn, Germany.

*Corresponding author

Tel: +49-228-73-69100, Fax: +49-228-73-69101, E-mail: bajorath@bit.uni-bonn.de

Supplementary Methods

Supplementary Results

Supplementary Figures S1-S3

Supplementary Table S1

Supplementary Methods

Compound selection

Bioactive compounds were extracted from ChEMBL version 26.[1] Only compounds with reported direct interactions (target relationship type: "D") with human targets at the highest confidence level (target confidence score: 9) and available exact activity measurements ("=") were pre-selected. Only standard potency measurements (K_i, IC_{50}, and K_d) were considered (and recorded as negative decadic logarithmic values). In addition, only compounds with potency values falling within the ($pK_i/pIC_{50}/pK_d$) range of 5 to 13 and having a molecular weight of less than 1000 Da were retained.

All compounds annotated as 'inactive', 'not active', 'inconclusive', 'potential transcription error', or 'pan assay interference compounds (PAINS)'[2] were discarded. Furthermore, all pre-selected compounds were required to pass the PAINS filter from RDKit,[3] a chemical liability filter covering medicinal chemistry rules,[4] and a filter from the Aggregator Advisor.[5] Compounds producing one or more alerts were discarded. In addition, known anti-targets and drug metabolizing enzymes were excluded. Filtering was carried out because for the assembly of compounds with multi-target activity, potential false positive activity assignments should be strictly avoided.

On the basis of selection criteria, 211,225 unique compounds with a total of 318,019 measurements for 1738 human targets were obtained and organized into 1738 target-based compound activity classes (target sets). Compound and activity data were extracted and standardized using in-house python scripts and with the aid of Open Eye chemistry toolkits.[6]

Machine learning

As a molecular representation, the Morgan fingerprint (with radius of 2)[7] from RDKit was calculated for each compound. As a control, the standard MACCS structural key (fragment) fingerprint[8] was used as an alternative molecular representation.

As machine learning methods, the random forest (RF)[9] and support vector machine (SVM)[10] algorithms were applied, as further discussed below.

In addition, a k-NN classifier (KNN) was generated. Therefore, training compounds were ranked based upon decreasing fingerprint similarity and the majority class label among the k top-ranked compounds was assigned to the test compound. Similarities were calculated on the basis of the Morgan fingerprint with a radius of 2 (folded into 2048-bit vector) from RDKit. The scikit-learn[11] implementation was used with default parameter settings (version 0.23.2), except for the number of NNs, for which optimal values were selected from k ∈ [1, 3, 5].

RF is a supervised ML algorithm generating an ensemble of independently derived decision trees using bootstrapping for random training data selection. The majority vote of all decision trees determines the predicted class label for test compounds. The scikit-learn RF implementation was used with default hyper-parameter settings (version 0.23.2), except for the number of decision trees ("n_estimators": 10, 100, 250, 500) and the minimum number of samples required to split an internal node ("min_samples_split": 2, 3, 5, 7, 10), which were optimized during training.

SVM is another supervised ML algorithm that constructs a hyperplane H in chemical feature space to best separate training instances with different class labels maximizing the margin between them. If linear separation is not possible in a given feature space, projection into higher-dimensional feature space is carried out where linear separation might be feasible. The projection is generated without explicit mathematical mapping through using kernel functions. The

relationship between training errors and margin size is controlled by the regularization hyper-parameter C, which was optimized using values of 0.1, 1, 10, 50, 100, 200, 400, 500, 750, and 1000. SVM classifiers were generated with scikit-learn using the Tanimoto[12] kernel. During hyper-parameter optimization, the best performing parameter was selected.

Control calculations using the MACCS fingerprint consistently produced similar results and the same clear trends, with overall marginally lower performance than the Morgan fingerprint. Therefore, results Morgan fingerprint are presented in the main manuscript.

Calculation protocols

For each prediction task, standard double cross-validation procedures were applied. First, during external cross validation, 50% of the compounds from each class were randomly selected and retained as a test set for model evaluation. External cross-validation was repeated over 10 independent trials with varying random seed values. During each trial, the remaining 50% of the compounds were used as a training set for model building and hyper-parameter optimization using 10 internal cross-validation trials (50:50 split). Performance measures were calculated as the average over 10 independent external cross-validation trials. Additionally, cross-pair predictions were carried out on corresponding test sets of remaining target pairs.

References

1. Gaulton A., Hersey A.; Nowotka M.; Bento A. P.; Chambers J.; Mendez D.; Mutowo P.; Atkinson F.; Bellis L. J.; Cibrián-Uhalte E.; Davies M.; Dedman N.; Karlsson A.; Magariños M. P.; Overington J. P.; Papadatos G.; Smit I.; Leach A. R. The ChEMBL database in 2017. *Nucleic Acids Res.* **2016**, *40*, D945–D954.
2. Baell, J. B.; Holloway, G. A. New Substructure Filters for Removal of Pan Assay Interference Compounds (PAINS) from Screening Libraries and for Their Exclusion in Bioassays. *J. Med. Chem.* **2010**, *53*, 2719–2740.
3. RDKit: Cheminformatics and Machine Learning Software, 2013. http://www.rdkit.org (accessed October 01, 2020).
4. Bruns, R. F.; Watson, I. A. Rules for Identifying Potentially Reactive or Promiscuous Compounds. *J. Med. Chem.* **2012**, *55*, 9763–9772.
5. Irwin, J. J.; Duan, D.; Torosyan, H.; Doak, A. K.; Ziebart, K. T.; Sterling, T.; Tumanian, G.; Shoichet, B. K. An Aggregation Advisor for Ligand Discovery. *J. Med. Chem.* **2015**, *58*, 7076–7087.
6. OEChem TK, version 1.7.7; OpenEye Scientific Software, Inc., Santa Fe, NM, USA, 2012.
7. Rogers, D.; Hahn, M. Extended-Connectivity Fingerprints. *J. Chem. Inf. Model.* **2010**, *50*, 742–754.
8. MACCS Structural Keys; Accelrys: San Diego, CA, 2011.
9. Breiman, L. Random Forests. *Mach. Learn.* **2001**, *45*, 5-32.
10. Vapnik, V. N. *The Nature of Statistical Learning Theory*, 2nd Ed.; Springer: New York, 2000.

11. Pedregosa, F.; Varoquaux, G.; Gramfort, A.; Michel, V.; Thirion, B.; Grisel, O.; Blondel, M.; Prettenhofer, P.; Weiss, R.; Dubourg, V.; Vanderplas, J.; Passos, A.; Cournapeau, D.; Brucher, M.; Perrot, M.; Duchesnay, E. Scikit-learn: Machine Learning in Python. *J. Mach. Learn. Res.* **2011**, *12*, 2825-2830.

12. Ralaivola, L.; Swamidass, S. J.; Saigo, H.; Baldi, P. Graph Kernels for Chemical Informatics. *Neural Netw.* **2005**, *18*, 1093–1110.

Supplementary Results

Figure S1. Cross-pair predictions of random samples. Boxplots (**a-h)** report results for eight of 10 random samples of cross-pair predictions (KNN: blue, RF: orange, SVM: green). The remaining two examples are shown in the main manuscript.

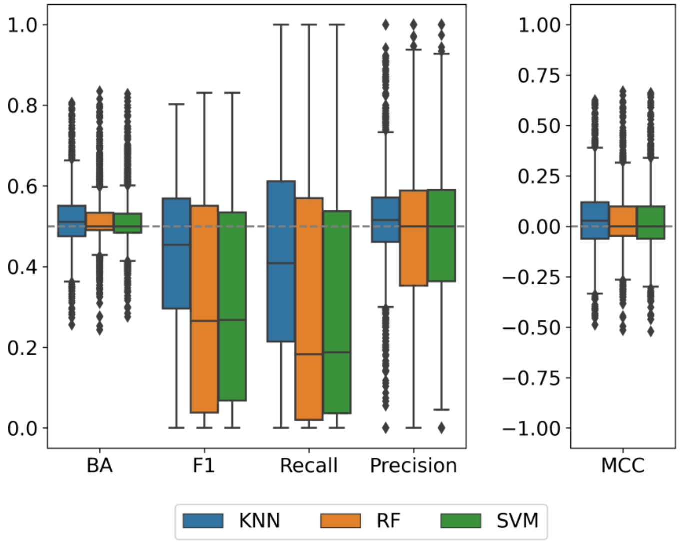

(a)

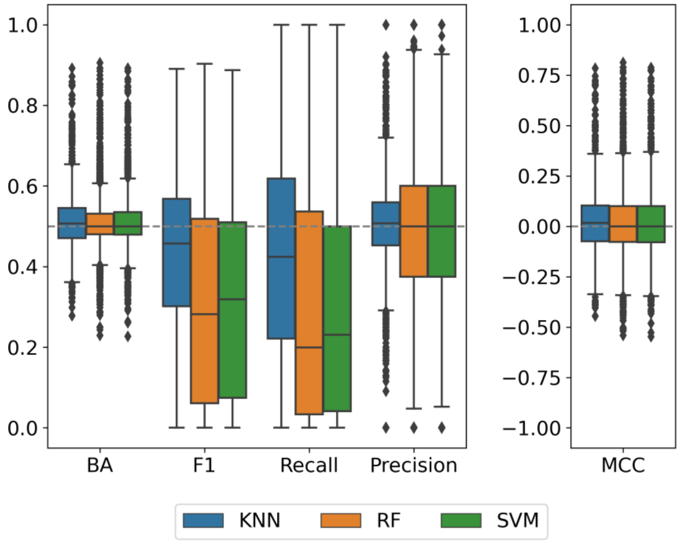

(b)

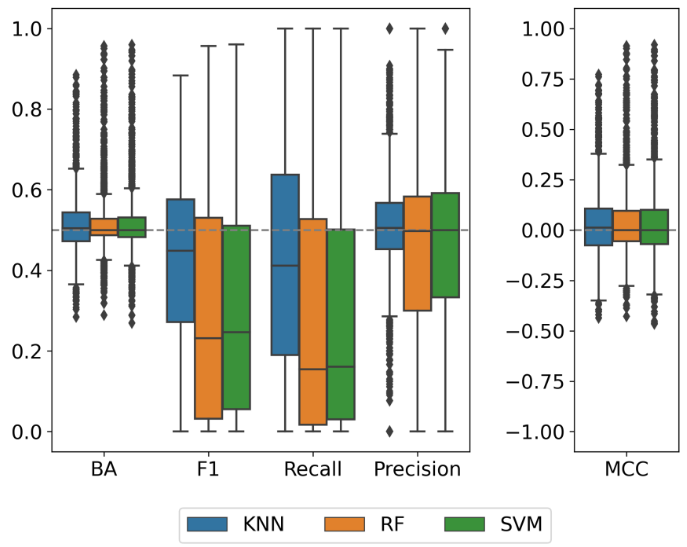
1.0
0.8
0.6
0.4
0.2
0.0
BA
F1
Recall
Precision
1.00
0.75
0.50
0.25
0.00
−0.25
−0.50
−0.75
−1.00
MCC
KNN
RF
SVM

(c)

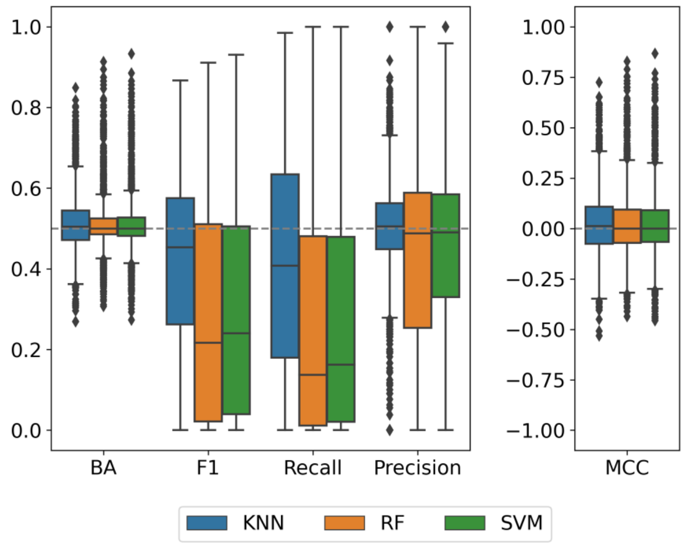
1.0
0.8
0.6
0.4
0.2
0.0
BA
F1
Recall
Precision
1.00
0.75
0.50
0.25
0.00
−0.25
−0.50
−0.75
−1.00
MCC
KNN
RF
SVM

(d)

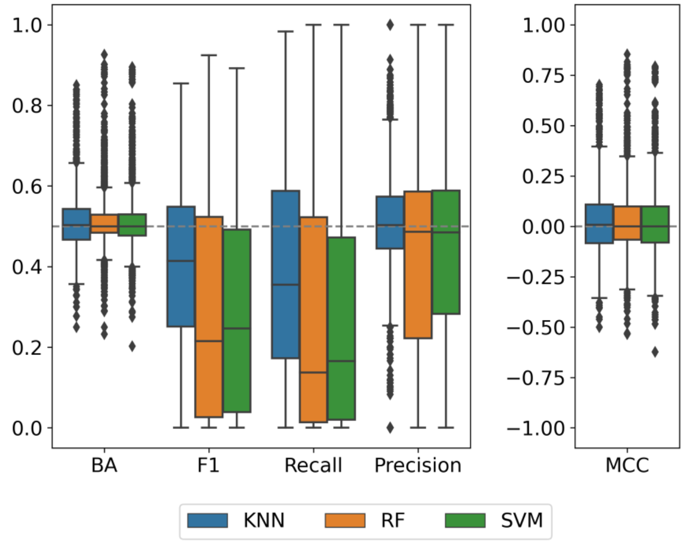
1.0
0.8
0.6
0.4
0.2
0.0
BA
F1
Recall
Precision
1.00
0.75
0.50
0.25
0.00
−0.25
−0.50
−0.75
−1.00
MCC
KNN
RF
SVM

(e)

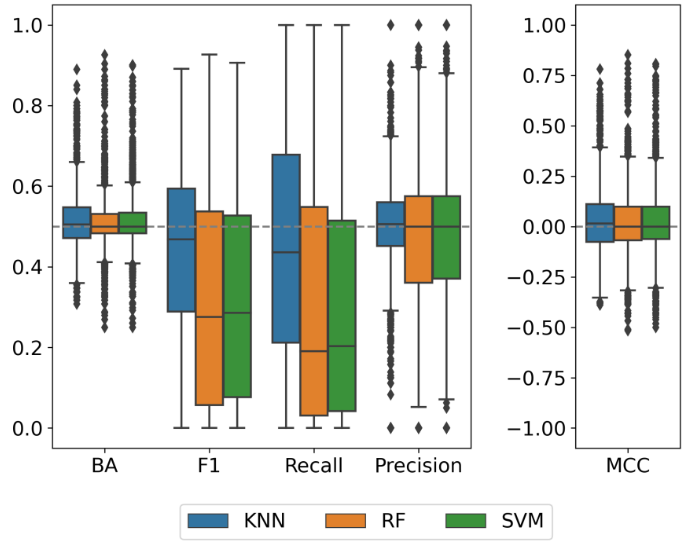
1.0
0.8
0.6
0.4
0.2
0.0
BA
F1
Recall
Precision
1.00
0.75
0.50
0.25
0.00
−0.25
−0.50
−0.75
−1.00
MCC
KNN
RF
SVM

(f)

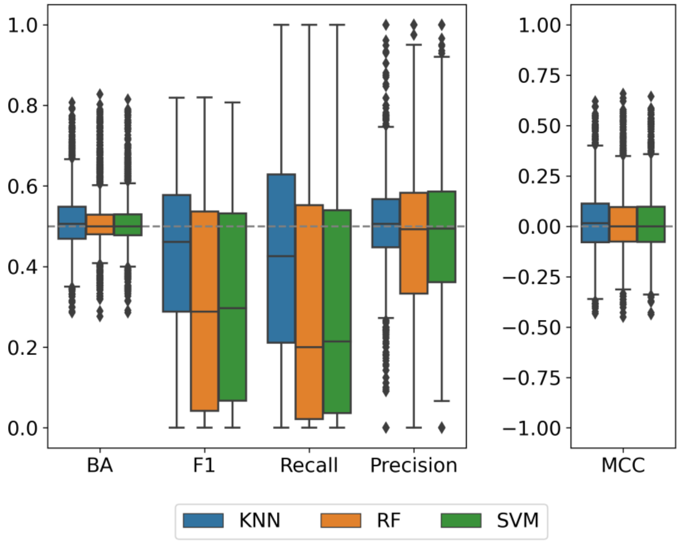
1.0
0.8
0.6
0.4
0.2
0.0
BA
F1
Recall
Precision
1.00
0.75
0.50
0.25
0.00
−0.25
−0.50
−0.75
−1.00
MCC
KNN
RF
SVM

(g)

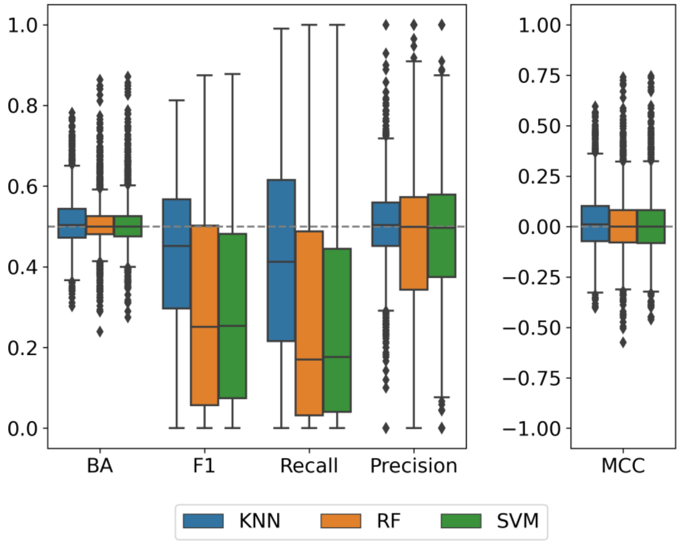
1.0
0.8
0.6
0.4
0.2
0.0
BA
F1
Recall
Precision
1.00
0.75
0.50
0.25
0.00
−0.25
−0.50
−0.75
−1.00
MCC
KNN
RF
SVM

(h)

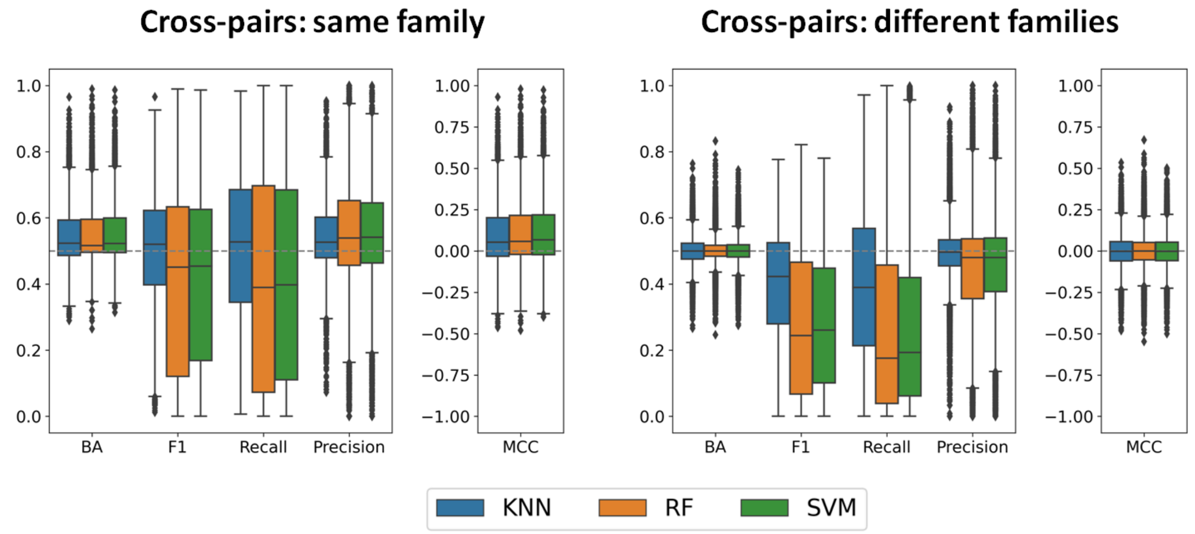

Figure S2. Cross-pair predictions for target pairs from the same family and different families. Cross-pairs of 157 target pairs from the same family were formed such that the four targets from both pairs belonged to the same family (5030 cross-pairs). In addition, cross-pairs were formed for target pairs belonging to different families (19,462 cross-pairs). Results of separate predictions for both cross-pair subsets are reported, revealing only minor differences.

Supplementary Table S1. Target pairs from different families. Listed are the UniProt ID, name, and family of targets forming pairs from different families.

Pair	Uniprot ID	Target name (target family)
A	O14684	Prostaglandin E synthase (MAPEG family)
A	P09917	Polyunsaturated fatty acid 5-lipoxygenase (Lipoxygenase family)
B	O14965	Aurora kinase A (Ser/Thr protein kinase family)
B	P23443	Ribosomal protein S6 kinase beta-1 (AGC Ser/Thr protein kinase family)
C	O14965	Aurora kinase A (Ser/Thr protein kinase family)
C	P35968	Vascular endothelial growth factor receptor 2 (Tyr protein kinase family)
D	P03956	Interstitial collagenase (Peptidase M10A family)
D	P78536	Disintegrin and metalloproteinase domain-containing protein 17 (None)
E	P08908	5-hydroxytryptamine receptor 1A (G-protein coupled receptor 1 family)
E	P31645	Sodium-dependent serotonin transporter (Sodium:neurotransmitter symporter (SNF) (TC 2.A.22) family)
F	P14416	D(2) dopamine receptor (G-protein coupled receptor 1 family)
F	P31645	Sodium-dependent serotonin transporter (Sodium:neurotransmitter symporter (SNF) (TC 2.A.22) family)
G	P14780	Matrix metalloproteinase-9 (Peptidase M10A family)
G	P78536	Disintegrin and metalloproteinase domain-containing protein 17 (None)
H	P23443	Ribosomal protein S6 kinase beta-1 (AGC Ser/Thr protein kinase family)
H	Q96GD4	Aurora kinase B (Ser/Thr protein kinase family)
I	P28223	5-hydroxytryptamine receptor 2A (G-protein coupled receptor 1 family)
I	P31645	Sodium-dependent serotonin transporter (Sodium:neurotransmitter symporter (SNF) (TC 2.A.22) family)
J	P28335	5-hydroxytryptamine receptor 2C (G-protein coupled receptor 1 family)
J	P31645	Sodium-dependent serotonin transporter (Sodium:neurotransmitter symporter (SNF) (TC 2.A.22) family)
K	P31645	Sodium-dependent serotonin transporter (Sodium:neurotransmitter symporter (SNF) (TC 2.A.22) family)
K	P34969	5-hydroxytryptamine receptor 7 (G-protein coupled receptor 1 family)
L	P31645	Sodium-dependent serotonin transporter (Sodium:neurotransmitter symporter (SNF) (TC 2.A.22) family)
L	Q9Y5N1	Histamine H3 receptor (G-protein coupled receptor 1 family)
M	P45452	Collagenase 3 (Peptidase M10A family)
M	P78536	Disintegrin and metalloproteinase domain-containing protein 17 (None)

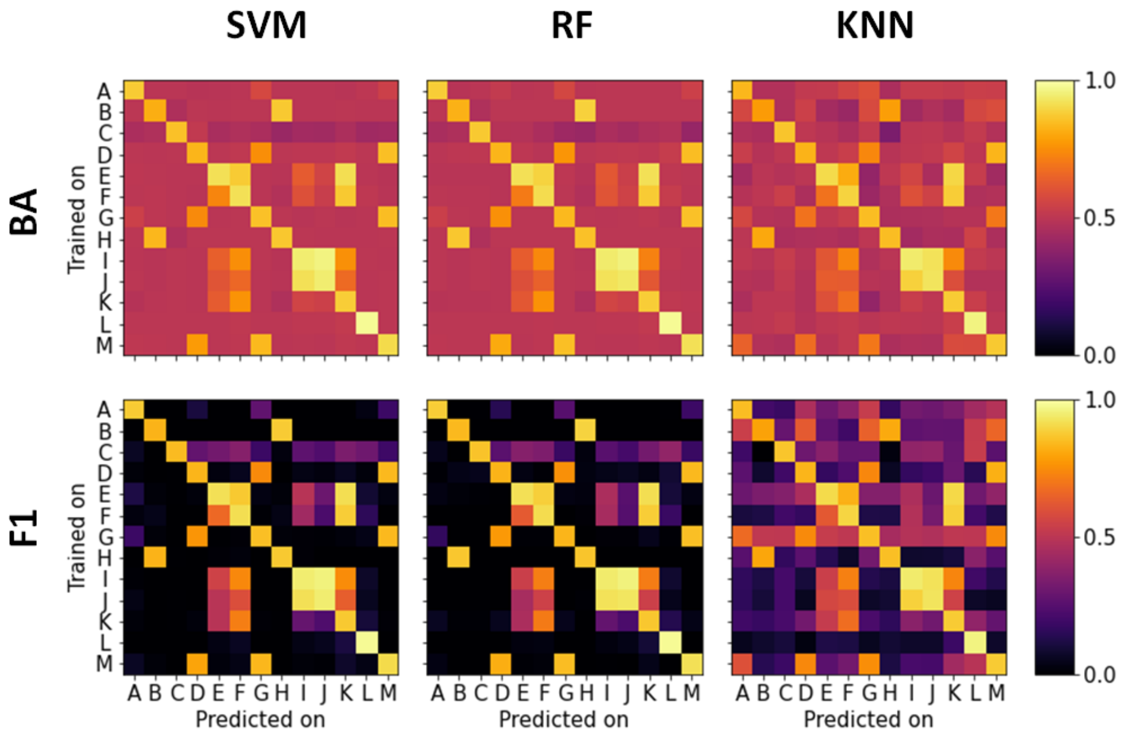

Figure S3. Prediction performance for pairs of targets from different families. Heatmaps report mean prediction accuracy (BA, F1) of models for pairs of targets from different families. Diagonal elements represent native predictions and off-diagonal elements cross-pair predictions.

Native predictions were highly accurate while most cross-pair predictions yielded random accuracy or worse. Among few cross-pair predictions with above-random accuracy was a highly accurate prediction (BA 0.92±0.03) of a model derived for target pair I (5-hydroxytryptamine receptor 2A, sodium-dependent serotonin transporter) and applied to classify compounds of pair J (5-hydroxytryptamine receptor 2C, sodium-dependent serotonin transporter), nearly reaching native performance for pair J (BA 0.96±0.03). This exception was attributable to a target correlation effect because pair I and J shared the sodium-dependent serotonin transporter target.
